# Assessment of Compliance With the National Institute for Health and Care Excellence Guidance in the Management of Patients Referred Following a First Suspected Seizure: A Retrospective Clinical Audit

**DOI:** 10.7759/cureus.118323

**Published:** 2026-10-21

**Authors:** Mohammed T Hazara, Zeenia Merali, Priyarenjini Sarasamma, Sinead Blake, Gibran Merali

**Affiliations:** 1 Internal Medicine, University Hospital Southampton NHS Foundation Trust, Southampton, GBR; 2 Paediatrics and Child Health, Royal National Orthopaedic Hospital NHS Trust, Watford, GBR; 3 Epilepsy, University Hospital Southampton NHS Foundation Trust, Southampton, GBR; 4 General Medicine, University Hospital Southampton NHS Foundation Trust, Southampton, GBR

**Keywords:** clinical audit, electroencephalography, epilepsy, first seizure, neurology, nice, quality improvement, seizure clinic

## Abstract

Introduction

A first suspected seizure requires timely specialist assessment and appropriate investigation to establish an accurate diagnosis, identify seizure mimics, assess the risk of recurrence, and provide appropriate safety advice. The National Institute for Health and Care Excellence (NICE) guidance recommends urgent specialist assessment following a first suspected seizure, alongside appropriate electrocardiography, electroencephalography, neuroimaging, recurrence-risk assessment, and patient safety advice. This audit evaluated adherence to these standards within a first-seizure clinic.

Materials and methods

A retrospective clinical audit was undertaken on patients referred to a first-seizure clinic from the Emergency Department and Acute Medicine Unit over a three-month period from January to March 2025. Data were obtained from electronic patient records, clinic correspondence, and discharge documentation. Compliance with predefined standards from NICE guidance was assessed. The primary outcome measures were timely specialist assessment, 12-lead electrocardiography, documentation of individualized seizure recurrence risk, timely electroencephalography where clinically indicated, timely neuroimaging where indicated, and documentation of safety-netting and driving advice.

Results

Sixty-three patient records were initially identified. Ten patients did not attend or were lost to follow-up and were excluded from the final clinical analysis, leaving 53 patients. Five of 53 patients (9.4%) received specialist assessment within two weeks. A 12-lead ECG was documented in all 53 patients (100%), and an individualized seizure recurrence risk assessment was documented in all patients (100%). An electroencephalogram (EEG) was indicated in 17 patients, of whom one (5.9%) underwent EEG within 72 hours; 15 (88.2%) underwent EEG after 72 hours, and one patient did not attend. The mean time to EEG among those who eventually underwent the investigation was approximately 12.5 weeks. Neuroimaging was indicated in 41 patients, of whom 22 (53.7%) underwent imaging within six weeks and 19 (46.3%) underwent imaging after six weeks. Safety-netting, including documentation of driving/Driver and Vehicle Licensing Agency (DVLA) advice, was documented in all 53 patients (100%).

Conclusion

The audit demonstrated high compliance with core assessment and documentation standards but substantial delays in specialist review and time-critical investigations. The greatest gap was in timely EEG access, followed by delays in specialist assessment and neuroimaging. The findings support service-level interventions aimed at improving urgent access to first-seizure assessment and investigations. A repeat audit, following the implementation of targeted interventions, will be important to determine whether pathway changes improve compliance.

## Introduction

A first suspected seizure is a common clinical presentation that requires timely assessment to establish whether the event was epileptic and to distinguish epilepsy from important seizure mimics such as syncope and other causes of transient loss of consciousness. Accurate diagnosis is clinically important because delays in specialist assessment and investigation may prolong diagnostic uncertainty and potentially delay appropriate management [1].

The National Institute for Health and Care Excellence (NICE) guideline NG217 recommends urgent referral for specialist assessment following a first suspected seizure, with an appointment within two weeks. NICE also recommends an individualized assessment of the risk of a second seizure and a 12-lead ECG because cardiac conditions may mimic epileptic seizures. Where an electroencephalogram (EEG) is requested following a first seizure, NICE recommends that it should be performed as soon as possible, ideally within 72 hours of the seizure. Neuroimaging is recommended when an underlying structural cause is suspected; MRI is recommended within six weeks of MRI referral for people diagnosed with epilepsy, subject to the guideline's specified exceptions [1].

The International League Against Epilepsy (ILAE) British Branch developed an Adult First Seizure Audit to support assessment of service provision, assessment quality, investigations, and counselling in secondary-care services [2,3]. The National Audit of Pathways in Epileptic Seizure Referrals (NAPIER), a multicenter audit of 727 patients across 25 hospitals in the United Kingdom and Ireland, demonstrated substantial delays in first-seizure services, with a median time to specialist review of 48 days and only 13.8% of patients assessed within two weeks [4]. The National Audit of Seizure Management in Hospitals (NASH) also demonstrated substantial variation in seizure assessment and management across UK hospitals, highlighting the importance of consistent pathways for patients presenting with seizures [5].

The diagnostic uncertainty associated with a first suspected seizure is substantial. In a prospective cohort of 200 adults referred to a first-seizure clinic, 181 patients attended, of whom 69 (38.1%) received a diagnosis other than epilepsy or first seizure, highlighting the importance of specialist assessment in distinguishing epileptic events from seizure mimics [6].

The clinical consequences of a first unprovoked seizure also depend on the individual's risk of recurrence. A quantitative review of 16 studies estimated an average recurrence risk of approximately 51% following a first unprovoked seizure, with recurrence at or near two years ranging from 36% to 47% in studies using first-seizure methodology [7]. More recent reviews have similarly estimated that approximately 40-50% of untreated patients experience a recurrent seizure within two years, with higher risk associated with factors such as an abnormal EEG or an underlying neurological abnormality [8]. Consequently, accurate assessment of recurrence risk is an important component of first-seizure management and informs decisions regarding investigation, counselling, and antiseizure medication.

Despite these recommendations, delays in specialist assessment remain common. NAPIER reported that 12.7% of patients experienced seizure recurrence while awaiting their first appointment, demonstrating the potential clinical consequences of delays in access to specialist assessment [4]. These findings suggest that timely access to specialist first-seizure services remains an important challenge within the UK healthcare system.

A local clinical audit provides an opportunity to identify gaps between recommended and delivered care and to develop targeted service-improvement interventions. The aim of this audit was therefore to evaluate the management of patients referred to a first-seizure clinic against predefined standards derived from NICE guidance and to identify areas for improvement within the local clinical pathway.

## Materials and methods

Study design and setting

A retrospective clinical audit was undertaken within a first-seizure clinic at a tertiary NHS hospital. The clinic receives referrals from the Emergency Department and Acute Medicine Unit for patients presenting with a suspected first seizure or a related transient loss of consciousness. The audit covered a three-month period from January to March 2025 and evaluated adherence to predefined care-process standards derived from relevant NICE guidance.

Study population

Patients referred to the first-seizure clinic from the Emergency Department and Acute Medicine Unit during the audit period were initially considered for inclusion. Patients who did not attend their appointment or were subsequently lost to follow-up were identified separately. These patients were excluded from the assessment of clinical management following specialist review but were retained in the overall pathway analysis. A total of 63 patient records were initially identified. Ten patients did not attend or were lost to follow-up, resulting in a final clinical analysis population of 53 patients.

Data sources

Data were obtained retrospectively from electronic patient records, first-seizure clinic documentation, clinic correspondence, and hospital discharge summaries. Variables included time from referral to specialist assessment, 12-lead ECG documentation, individualized seizure recurrence risk assessment, EEG performance and timing where indicated, neuroimaging performance and timing where indicated, safety-netting and driving/Driver and Vehicle Licensing Agency (DVLA) advice, and the final documented clinical diagnosis.

The indications for EEG and neuroimaging were determined by the clinical judgement of the healthcare professionals involved in each patient's assessment and management, informed by the clinical presentation and NICE guidance.

Audit standards

Audit standards were developed from relevant NICE recommendations concerning assessment following a first suspected seizure. The predefined standards were specialist assessment within two weeks; a 12-lead ECG performed and documented; an individualized seizure recurrence risk assessment documented; a timely EEG where clinically indicated, interpreted in line with the NICE recommendation that EEGs should be performed as soon as possible and ideally within 72 hours of the seizure; and timely neuroimaging where clinically indicated, with reference to NICE, which recommends MRI within six weeks of referral for eligible patients diagnosed with epilepsy. NICE further recommends CT imaging when MRI is contraindicated and documentation of safety-netting advice, including driving/DVLA advice where appropriate.

Data analysis

Data were analyzed descriptively. Compliance with each audit standard was calculated as the proportion of eligible patients meeting the predefined criterion. For investigations that were only indicated in a subgroup, compliance was calculated using the number of eligible patients as the denominator. EEG timing was assessed using the seizure occurrence documented in the Emergency Department records and the date of EEG performance. As this was a retrospective audit of routine clinical practice, the analysis relied on information documented in the available clinical records, and no additional retrospective criteria were applied to determine the original clinical indications for investigation. No inferential statistical testing was undertaken because the primary purpose of the project was service evaluation and identification of quality-improvement opportunities.

Governance and ethical considerations

As this project was classified as a retrospective clinical audit/service evaluation of existing practice, individual patient consent and NHS Research Ethics Committee approval were not required. The project was conducted under local Trust clinical governance approval.

## Results

The patient flow is shown in Figure 1. Sixty-three patient records were identified during the three-month audit period. Ten patients (15.9%) did not attend their appointments or were lost to follow-up and were excluded from the final clinical analysis. The final analysis therefore included 53 patients.

**Figure 1:**
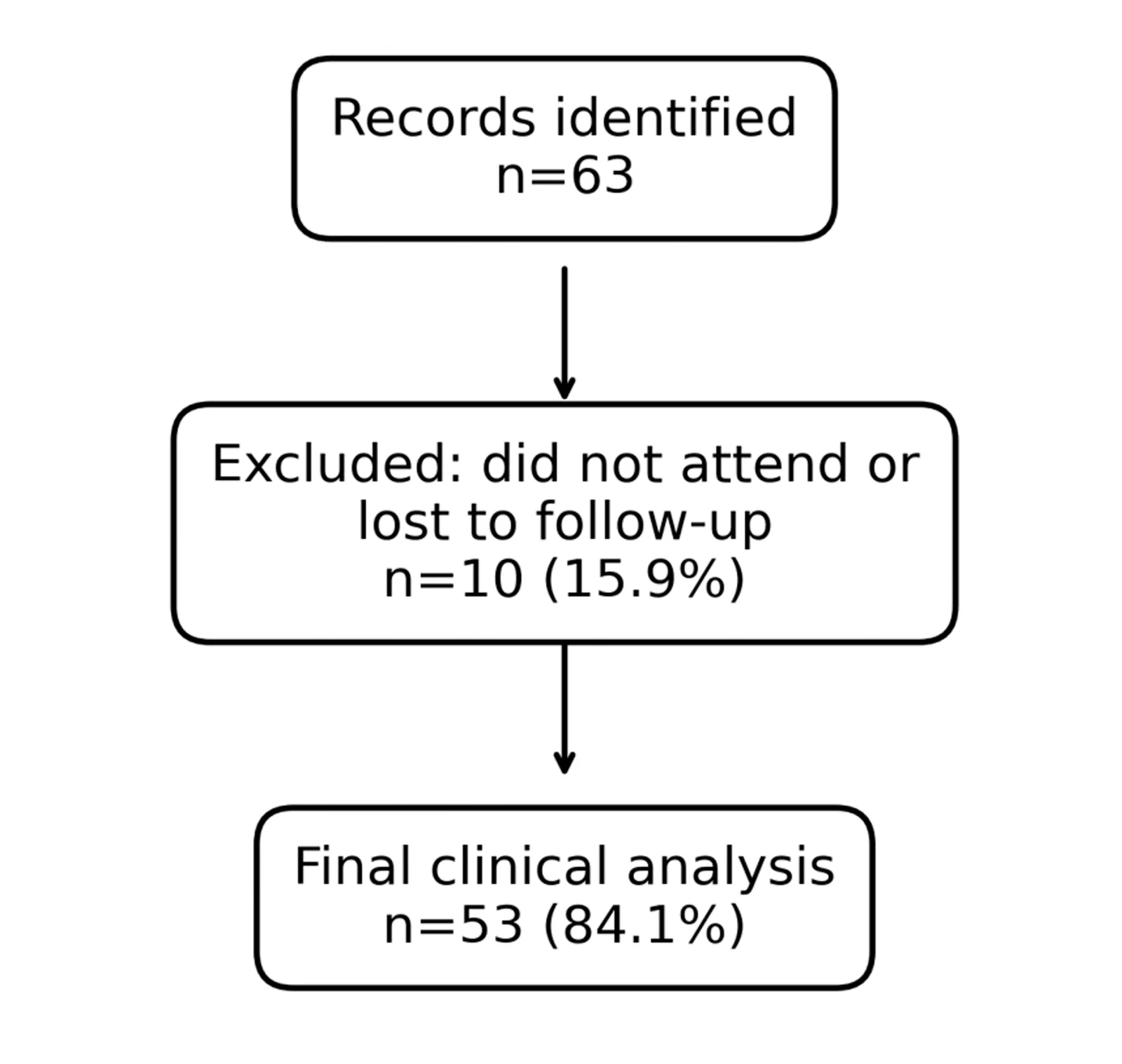
Patient flow through the audit.

Specialist assessment

Only five of 53 patients (9.4%) were assessed by a specialist within two weeks. The remaining patients were assessed after two weeks, with substantial variation in waiting time. The distribution of time from referral to specialist assessment is presented in Table 1. Specialist assessment occurred as late as six months after referral in one patient. Two patients experienced particularly prolonged waits of four and six months after missing their original appointments.

**Table 1:** Time from referral to specialist assessment.

Time for specialist assessment	Number of patients (n)	% of cohort (n = 53)
Within 2 weeks	5	9.4%
4 weeks	7	13.2%
5 weeks	3	5.7%
6 weeks	8	15.1%
6.5 weeks	1	1.9%
7 weeks	5	9.4%
7.5 weeks	1	1.9%
8 weeks	7	13.2%
8.5 weeks	1	1.9%
9 weeks	5	9.4%
9.5 weeks	1	1.9%
10 weeks	3	5.7%
11 weeks	1	1.9%
12 weeks	2	3.8%
15 weeks	1	1.9%
4 months	1	1.9%
6 months	1	1.9%
Total	53	100%

12-lead ECG and recurrence-risk assessment

A 12-lead ECG was performed and documented in all 53 patients (100%). Documentation of an individualized assessment of seizure recurrence risk was present in all 53 patients (100%).

EEG

EEG was considered indicated in 17 of the 53 patients. The timing and outcomes of EEG assessments among these patients are summarized in Table 2. One of the 17 patients (5.9%) underwent EEG within 72 hours. Fifteen patients (88.2%) underwent EEG after 72 hours, while one patient (5.9%) did not attend. Among patients who underwent EEG after the recommended timeframe, the mean time to EEG was approximately 12.5 weeks, with a range from four weeks to six months. The single EEG completed within 72 hours was performed during an inpatient admission.

**Table 2:** Time to electroencephalogram (EEG) among patients with an indication (n = 17).

EEG outcome	n	% of indicated EEGs
Within 72 hours	1	5.9%
4 weeks	1	5.9%
8 weeks	1	5.9%
9 weeks	2	11.8%
10 weeks	2	11.8%
11 weeks	1	5.9%
12 weeks	1	5.9%
13 weeks	2	11.8%
15 weeks	1	5.9%
16 weeks	2	11.8%
17 weeks	1	5.9%
6 months	1	5.9%
Did not attend	1	5.9%
Total	17	100%

Neuroimaging

Neuroimaging was considered indicated in 41 of the 53 patients (77.4%). The timing of neuroimaging among patients with an indication is presented in Table 3. Twenty-two of these 41 patients (53.7%) underwent imaging within six weeks, while 19 patients (46.3%) underwent imaging after six weeks. The time to imaging ranged from same-day imaging to 20 weeks.

**Table 3:** Time to neuroimaging among patients with an indication (n = 41).

Time to neuroimaging	n	% of indicated imaging
Same day	4	9.8%
1 day	3	7.3%
2 days	2	4.9%
3 days	1	2.4%
4 days	1	2.4%
6 days	1	2.4%
2 weeks	1	2.4%
4 weeks	2	4.9%
5 weeks	2	4.9%
6 weeks	5	12.2%
7 weeks	4	9.8%
8 weeks	5	12.2%
9 weeks	2	4.9%
10 weeks	3	7.3%
11 weeks	1	2.4%
12 weeks	2	4.9%
13 weeks	1	2.4%
20 weeks	1	2.4%
Total	41	100%

Safety-netting

Safety-netting documentation, including driving/DVLA advice and patient information where appropriate, was present in all 53 patients (100%).

Final documented diagnosis

The most frequent final documented diagnosis was tonic-clonic seizure, accounting for 19 of 53 patients (35.8%). Syncope was documented in 13 patients (24.5%), transient loss of consciousness in six patients (11.3%), and alcohol-related events in five patients (9.4%). The complete distribution of final documented diagnoses and events is shown in Table 4. Other diagnoses included dissociative seizure, non-epileptic seizure, significant amnesia, focal seizure, and intermittent left-hand numbness.

**Table 4:** Final documented diagnoses/events.

Diagnosis/event	n	% of cohort
Tonic-clonic seizure	19	35.8%
Syncope	13	24.5%
Transient loss of consciousness	6	11.3%
Alcohol-related event	5	9.4%
Dissociative seizure	4	7.5%
Non-epileptic seizure	3	5.7%
Significant amnesia	1	1.9%
Focal seizure	1	1.9%
Left-hand intermittent numbness	1	1.9%
Total	53	100%

Overall compliance with the predefined audit standards is summarized in Table 5. Compliance was 100% for 12-lead ECG documentation, individualized seizure recurrence-risk assessment, and safety-netting, including DVLA advice where appropriate. Compliance was substantially lower for specialist assessment within two weeks (9.4%) and EEG within 72 hours where indicated (5.9%), while neuroimaging within six weeks where indicated was achieved in 53.7% of patients.

**Table 5:** Overall compliance with predefined audit standards.

Audit standard	Compliant	Denominator	Compliance
Specialist review within two weeks	5	53	9.4%
12-lead ECG performed and documented	53	53	100.0%
Individualized seizure recurrence risk assessment	53	53	100.0%
EEG within 72 hours, where indicated	1	17	5.9%
Neuroimaging within six weeks, where indicated	22	41	53.7%
Safety-netting, including DVLA advice, where appropriate	53	53	100.0%

## Discussion

This retrospective clinical audit demonstrated marked variation in compliance across different components of the first-seizure pathway. Documentation-based standards were achieved consistently, with 100% compliance for 12-lead ECG documentation, individualized seizure recurrence-risk assessment, and safety-netting, including driving/DVLA advice. In contrast, time-dependent standards demonstrated substantially lower compliance, particularly with timely specialist assessment and EEG, while neuroimaging showed intermediate compliance. The principal deficiencies identified were therefore related to the timeliness of specialist assessment and investigations rather than documentation of clinical assessment, risk assessment, or safety-netting.

Only five of 53 patients (9.4%) were assessed by a specialist within two weeks of referral. This compares with NAPIER, a multicenter audit of 727 patients across 25 hospitals in the United Kingdom and Ireland, in which the median time to specialist review was 48 days, and only 13.8% of patients were assessed within two weeks [4]. NAPIER also reported that 12.7% of patients experienced seizure recurrence while awaiting their first appointment [4]. The similarity between the present findings and the national audit suggests that delays in accessing specialist first-seizure assessment may reflect broader organizational and service-capacity challenges rather than an isolated local problem. Delayed specialist assessment may prolong diagnostic uncertainty, delay appropriate investigation and management, and postpone counselling regarding safety, driving, and recurrence risk. These findings therefore support the importance of maintaining a clearly prioritized pathway for patients with suspected first seizures in accordance with NICE recommendations [1].

The findings are also consistent with earlier UK national audit data demonstrating substantial variation in the assessment and onward management of patients presenting with seizures. NASH included 4,544 seizure-related attendances across 154 emergency departments and identified substantial variation in assessment and referral practices [5]. In comparison, the present audit achieved 100% documentation of safety-netting and driving/DVLA advice. Although differences in methodology and clinical setting limit direct comparison, this represents an area of particularly strong local performance. It suggests that, once patients entered the specialist pathway, important aspects of patient-safety counselling were consistently documented.

EEG represented the lowest compliance among the time-dependent investigation standards. Of the 17 patients for whom EEG was considered indicated, only one patient (5.9%) underwent EEG within 72 hours, while 15 (88.2%) underwent EEG after 72 hours and one patient (5.9%) did not attend. Among patients who underwent EEG after the recommended timeframe, the mean time to EEG was approximately 12.5 weeks, with a range from four weeks to six months. The single EEG completed within 72 hours was performed during an inpatient admission, suggesting that rapid access was achievable when EEG could be arranged within an acute-care setting but was considerably more difficult when dependent on routine outpatient scheduling.

NICE NG217 recommends that, when an EEG is requested following a first seizure, it should be performed as soon as possible, ideally within 72 hours of the seizure [1]. The present findings therefore identify rapid EEG access as a major area for improvement. The interpretation of this finding requires some caution when comparing it with NAPIER because the two audits use different timing measures. NAPIER reported a median time to EEG of 30 days among patients with epilepsy, whereas the present audit calculated the interval from the documented seizure onset to EEG performance [4]. The measures are therefore not directly equivalent. Nevertheless, both audits demonstrate that timely access to EEG remains challenging within first-seizure pathways. The fact that the only EEG meeting the 72-hour standard in the present audit was performed during an inpatient admission further suggests that rapid access may be possible when neurophysiology can be arranged within an acute-care pathway.

Evidence from established first-seizure services demonstrates that more timely investigation is achievable. McIntosh et al. reviewed 1,555 patients diagnosed with new-onset unprovoked seizures or epilepsy across two established first-seizure clinics in Australia [9]. Around 86% of patients attended the first-seizure clinic within three weeks of the index seizure, 73% underwent EEG within three weeks, and 76% underwent MRI within six weeks. Epileptiform discharges were identified in 25% of patients undergoing EEG, while potentially epileptogenic abnormalities were identified in 19% of patients undergoing imaging [9]. Although the Australian setting and study population differ from the present audit, these findings demonstrate the potential for established first-seizure services to coordinate specialist assessment and investigations within relatively short timeframes. The substantially longer EEG waiting times observed locally therefore represent an important opportunity for service improvement.

Neuroimaging demonstrated better, although incomplete, compliance than EEG. Of the 41 patients considered to have an indication for neuroimaging, 22 (53.7%) underwent imaging within six weeks, while 19 (46.3%) underwent imaging after six weeks. The time to imaging ranged from same-day imaging to 20 weeks. This distribution suggests that imaging performed during the acute presentation was generally more timely than imaging arranged subsequently through outpatient pathways, where patients were dependent on routine scheduling.

The 53.7% compliance observed in the present audit compares with the NAPIER findings, in which the median time to MRI among patients with epilepsy was 37 days [4]. The difference in reporting format means that the two results cannot be directly compared as percentages meeting a six-week threshold. The Australian first-seizure clinic cohort reported by McIntosh et al. found that 76% of patients underwent MRI within six weeks [9]. The lower proportion achieving the six-week timeframe in the present audit may reflect differences in imaging capacity, referral pathways, patient selection, and local organizational structures. Nevertheless, the finding indicates that coordination between specialist assessment, imaging referral, and radiology scheduling represents another potential area for service improvement.

The diagnostic profile of the present cohort further demonstrates the importance of specialist assessment following a suspected first seizure. Although tonic-clonic seizure was the most frequent documented diagnosis, a substantial proportion of patients were ultimately considered to have alternative diagnoses or events, including syncope, transient loss of consciousness, alcohol-related events, dissociative seizures, and non-epileptic seizures. This is consistent with previous first-seizure clinic literature demonstrating substantial diagnostic uncertainty among referred patients. Mc Manus et al. reported that, among patients referred to a first-seizure clinic, a substantial proportion received diagnoses other than epilepsy or remained diagnostically indeterminate [6]. Similarly, McFadyen's prospective audit of 200 consecutive first-seizure clinic attendees in Glasgow found that 28% were newly diagnosed with epilepsy and 13% with an unprovoked first seizure, with alcohol- and/or drug-related factors contributing to a further proportion of seizure presentations [10]. These findings reinforce that referral for a suspected first seizure does not equate to a diagnosis of epilepsy and that specialist assessment has an important role in distinguishing epileptic seizures from syncope and other seizure mimics.

The need for individualized assessment is also supported by the established literature on recurrence following a first unprovoked seizure. Recurrence risk is not uniform and varies according to clinical characteristics and investigation findings [7,8]. The 100% documentation rate for individualized recurrence-risk assessment in the present audit is therefore an important finding, as it indicates that risk stratification was consistently incorporated into the specialist assessment despite the delays observed elsewhere in the pathway.

The 15.9% rate of non-attendance or loss to follow-up represents another important service-level finding. Although these patients were excluded from the final clinical analysis because their subsequent management could not be assessed, non-attendance itself represents a potential vulnerability within a time-sensitive diagnostic pathway. NAPIER reported that participating hospitals had substantial numbers of patients who did not attend their first-seizure clinic appointments, with an average DNA rate of 17.2% among hospitals providing this information [4]. This is comparable with the 15.9% rate observed in the present audit. McFadyen's prospective Glasgow audit also reported a 16% non-attendance rate for first-seizure clinic review appointments [10]. Taken together, these findings suggest that non-attendance is a recurring challenge across first-seizure services rather than an isolated local phenomenon. In the present audit, two patients experienced particularly prolonged waits after missing their original appointments, highlighting the potential consequences when missed appointments are not rapidly identified and rebooked. Active follow-up of non-attenders may therefore represent a practical intervention to reduce loss from the diagnostic pathway.

The findings also support consideration of service redesign rather than solely clinician-level interventions. A UK quality-improvement project examining access to first-seizure clinics following emergency-department attendance reduced the median waiting time from 65 days before intervention to 31 days following implementation of a structured improvement program [11]. Although this represented a significant improvement, the service did not achieve the NICE recommendation of assessment within 14 days [11]. This experience is particularly relevant to the present audit because it demonstrates that streamlining referral processes can produce meaningful reductions in waiting times but may be insufficient to achieve the recommended target if specialist clinic capacity remains constrained. The present findings similarly suggest that both pathway redesign and sufficient specialist capacity may be required to achieve sustained improvement.

Several service-level interventions could therefore be considered. Referral triage could be reviewed to ensure that suspected first-seizure referrals are appropriately prioritized for specialist assessment within two weeks, with a standardized referral mechanism from the Emergency Department and Acute Medicine Unit. Additional dedicated first-seizure clinic sessions or appropriately trained seizure specialists could be considered where referral demand exceeds available capacity. Given that only one of 17 patients underwent EEG within 72 hours, a protected urgent EEG pathway or rapid-access EEG slots could also be explored. Improved coordination between specialist assessment, EEG, and neuroimaging services may further reduce delays associated with separate outpatient booking processes. Finally, an active follow-up and rebooking system for patients who do not attend their initial appointment or investigation could reduce the risk of patients being lost from a time-sensitive diagnostic pathway.

Taken together, these findings suggest that the local service performs well in areas of clinical documentation and patient counselling but less well in time-dependent aspects of care. This distinction is important because the findings do not indicate a general failure to implement guideline-based practice. Instead, they suggest that once patients enter the specialist pathway, key elements of assessment, recurrence-risk documentation and safety-netting are consistently recorded, whereas access to specialist review and investigations is constrained by pathway and capacity issues. This interpretation is consistent with NAPIER, which demonstrated considerable variation between centers and identified delays in access to specialist assessment and investigation across first-seizure services [4]. The NASH audit similarly identified wide variation in seizure management between hospitals [5]. The present audit therefore adds local evidence to a broader body of literature indicating that service organization is an important determinant of the quality and timeliness of first-seizure care.

A repeat audit following implementation of service changes would provide an opportunity to determine whether these interventions improve compliance. The same definitions and denominators should be retained where possible to permit direct comparison between audit cycles. Future measurement should include the proportion of patients reviewed within two weeks, the interval from documented seizure onset to EEG, the interval from MRI referral to imaging, and the proportion of referred patients who do not attend. A closed-loop audit approach would allow the service to determine whether changes in clinic capacity, referral triage, and access to investigations result in measurable improvement.

Strengths and limitations

A strength of this audit is that it examined multiple stages of the first-seizure pathway rather than focusing on a single investigation or outcome. This allowed areas of good practice to be identified alongside specific deficiencies requiring improvement. The inclusion of referrals from both the Emergency Department and Acute Medicine Unit also provided information across more than one referral source and captured different routes into the specialist pathway.

The retrospective, single-center design and relatively small sample size limit the generalisability of the findings. Local referral volumes, clinic capacity, availability of neurophysiology services, and access to neuroimaging may differ substantially between institutions. Consequently, the findings should primarily be interpreted as a service evaluation of local practice rather than as evidence of national performance. Nevertheless, comparison with NAPIER and other first-seizure clinic literature suggests that the principal areas of difficulty identified locally are also recognized within wider first-seizure services [4,9-11].

Retrospective audits are dependent on the quality and completeness of electronic documentation. Absence of documentation does not necessarily prove that an intervention did not occur. However, documented evidence remains important from a clinical governance perspective because it demonstrates that recommended care was communicated and supports continuity of care between clinicians. This is particularly relevant for safety-netting and driving advice, where failure to document counselling may make it difficult to establish whether appropriate advice was provided.

Ten patients did not attend or were lost to follow-up. Excluding these patients from the final clinical analysis was necessary because subsequent specialist assessment and investigation could not be reliably evaluated. However, this approach may underestimate problems within the overall referral pathway because patients who do not attend may remain without diagnostic clarification or appropriate safety advice. Their non-attendance was therefore retained as a separate service-level outcome. Future audit cycles should report both the overall referral cohort and the evaluable clinical cohort so that losses to follow-up remain visible.

Finally, investigation timing was dependent on the clinical information available within the electronic patient record. For EEG, the interval was calculated from the documented time of seizure onset to the time of EEG performance, allowing assessment against the NICE recommendation that EEG should be performed as soon as possible, ideally within 72 hours of the seizure [1]. This measure depends on accurate documentation of seizure onset and may be affected when the onset time is uncertain or retrospectively reported. For neuroimaging, compliance was assessed among patients for whom imaging was considered clinically indicated. Although NICE recommends MRI within six weeks of referral for eligible people diagnosed with epilepsy, this timeframe does not apply universally to all patients following a first suspected seizure. Future audit cycles should therefore record the imaging modality, MRI referral date, epilepsy diagnosis status, and relevant NICE eligibility criteria to enable more precise assessment of guideline adherence [1]. These differences in timing definitions should be considered when comparing the present findings with other audits, including NAPIER, and emphasise the importance of using clearly defined and reproducible measures in future audit cycles.

## Conclusions

This clinical audit identified high compliance with several core elements of first-seizure assessment, including 12-lead ECG documentation, individualized seizure recurrence risk assessment, and safety-netting and driving advice. However, substantial delays were identified in specialist assessment and investigations. Only 9.4% of patients were assessed within two weeks, 5.9% of patients with an indication for EEG underwent the investigation within the audit's 72-hour threshold, and 53.7% of patients considered to have an indication for neuroimaging underwent imaging within six weeks. The principal area for improvement is timely access to specialist assessment and diagnostic investigations, particularly EEG. Targeted interventions addressing referral prioritization, rapid-access EEG, imaging pathways, and active follow-up of patients who do not attend may improve compliance with recommended standards. A repeat audit following implementation of these interventions is warranted to determine whether pathway changes result in measurable improvement.
